# Protective Effects of Methylsulfonylmethane on Hemodynamics and Oxidative Stress in Monocrotaline-Induced Pulmonary Hypertensive Rats

**DOI:** 10.1155/2012/507278

**Published:** 2012-10-15

**Authors:** Sadollah Mohammadi, Moslem Najafi, Hossein Hamzeiy, Nasrin Maleki-Dizaji, Masoud Pezeshkian, Homayon Sadeghi-Bazargani, Masoud Darabi, Sara Mostafalou, Shahab Bohlooli, Alireza Garjani

**Affiliations:** ^1^Department of Pharmacology and Toxicology, School of Pharmacy, Tabriz University of Medical Sciences, Tabriz 5165665931, Iran; ^2^Division of Molecular Toxicology, Institute of Environmental Medicine, Karolinska Institutet (KI), Box 210, 17177 Stockholm, Sweden; ^3^Department of Pharmacology and Physiology, School of Medicine, Ardabil University of Medical Sciences, Ardabil 56135665, Iran; ^4^Research Center for Pharmaceutical Nanotechnology (RCPN), Tabriz University of Medical Sciences, Tabriz 5165691749, Iran; ^5^Drug Applied Research Center (DARC), Tabriz University of Medical Sciences, Tabriz 5165665811, Iran; ^6^Department of Cardiac Surgery and Cardiovascular Research Center, School of Medicine, Tabriz University of Medical Sciences, Tabriz 5166615573, Iran; ^7^Department of Statistics and Epidemiology, School of Health and Nutrition, Tabriz University of Medical Sciences, Tabriz 5166614711, Iran; ^8^Department of Biochemistry and Clinical Laboratories, School of Medicine, Tabriz University of Medical Sciences, Tabriz 5166615731, Iran; ^9^Department of Toxicology, Pharmaceutical Sciences Research Center (PSRC) and School of Pharmacy, Tehran University of Medical Sciences, Tehran 141556451, Iran

## Abstract

Methylsulfonylmethane (MSM) is naturally occurring organic sulfur that is known as a potent antioxidant/anti-inflammatory compound. The aim of this study was to investigate the effect of MSM on hemodynamics functions and oxidative stress in rats with monocrotaline- (MCT-) induced pulmonary arterial hypertension (PAH). Wistar rats were randomly assigned to 38-days treatment. MSM was administered to rats at 100, 200, and 400 mg/kg/day doses 10 days before a single dose of 60 mg/kg, IP, MCT. Hemodynamics of ventricles were determined by Powerlab AD instrument. Blood samples were obtained to evaluate changes in the antioxidative system including activities of catalase (CAT), superoxide dismutase (SOD), glutathione peroxidase (GPx), and the level of reduced glutathione (GSH) and malondialdehyde (MDA). Improvements in cardiopulmonary hemodynamics were observed in the MSM-treated pulmonary arterial hypertensive rats, with a significant reduction in right ventricular systolic pressure (RSVP) and an increase in the mean arterial pressure (MAP). The values of CAT, SOD, GSH-px activities, and GSH were significantly lower in MCT-induced PAH (*P* < 0.01), but they were recovered to control levels of MSM-treated groups. Our present results suggest that long-term administration of the MSM attenuates MCT-induced PAH in rats through modulation of oxidative stress and antioxidant defense.

## 1. Introduction

Pulmonary arterial hypertension (PAH) is a pathophysiological state characterized by a progressive increase in pulmonary vascular resistance. In addition to inducing myocardial hypertrophy, it also induces marked interstitial fibrosis to compensate for the increased ventricular workload. These adaptive changes often clinically lead to heart failure and sudden cardiac death [1]. Despite all current progress in the diagnosis and therapeutics, PAH continues to be a devastating disease with a high morbidity and mortality [2].

Recent reports have implicated increased oxidative stress as a mediator in the pathogenesis and the development of PAH [3]. Accordingly, antioxidant therapy has been effective in the treatment of right ventricle (RV) dysfunction in PAH [4].

Methylsulfonylmethane (MSM) is naturally occurring organic sulfur that is known as a potent antioxidant/anti-inflammatory compound [5, 6]. MSM is widely used as an arthritis remedy with potential anti-inflammatory effects [7, 8]. MSM may also be beneficial in PAH due to its anti-inflammatory and antiproliferative effects [9]. Despite an increasing clinical use, the mechanisms by which MSM exerts its effects remain largely unknown.

Monocrotaline (MCT) is a toxic pyrrolizidine alkaloid and has a selective toxic effect on pulmonary vessels without an effect on systemic vessels. Recently, we have observed that the expression of oxidative stress-related substances such as angiotensin II and endotheline 1 is increased in rats after exposure to MCT and that treatment with MSM suppresses these responses (unpublished findings). MSM was shown to act directly as free radical scavenger, which would further add to the efficiency of MSM as an antioxidant [7]. Thus, it is possible that MSM exerts its effect on PAH by interfering with oxidative events that may be associated with the heart failure. To investigate whether MSM may provide preventive effects on PAH, an experimental study was conducted examining the activity of antioxidative enzymes, including superoxide dismutase (SOD), catalase (CAT), and glutathione peroxidase (GSH-Px) in the serum samples from rats with MCT-induced PAH subjected to pretreatment. Moreover, we analyzed the nonenzymatic antioxidants reduced glutathione (GSH)/oxidized glutathione (GSSG) and the levels of malondialdehyde (MDA) as a lipid peroxidation biomarker.

## 2. Methods and Materials

### 2.1. Animals

Two-month-old male Wistar rats (200 ± 20 g) were, fed with standard laboratory chow ad libitum, used in the experiment, as previously described by us [10]. Animals were obtained from the Pasteur Institute of Iran (Tehran, Iran). Tabriz University of Medical Sciences Animal Ethics Committee approved the study protocol. Injections were all administered intraperitoneally (i.p.) to rats. Invasive experimental procedures were carried out on pentobarbital anaesthetized rats (60 mg/kg body weight, i.p.). At the end of the experiment, blood samples were obtained from the inferior vena cava under general anesthesia [11] for determination of serum SOD, GPx, GSH, and CAT. Then animals were sacrificed by pentobarbital overdose [12], and heart and lung tissues were excised to calculate tissue wet-to-body and wet-to-dry weight ratios.

### 2.2. Experimental Protocol

PAH was induced by means of a single dose of MCT (Sigma-Aldrich; 60 mg/kg) [13]. The effective doses of MSM (Fluka/Sigma-Aldrich) were determined according to a significant improvement in hemodynamic status of MCT-induced PAH rats [14]. Rats were subjected to treatment with MSM (0–400 mg/kg/day) 10 days before MCT injection (*n* = 48) and continued until 4 weeks after the MCT injection. Saline was used as vehicle in control experiments. MSM was well tolerated by the rats and no abnormal behavior was observed.

### 2.3. Hemodynamic Measurements

All hemodynamic measurements were carried out in rats, as described previously [10]. Right ventricular systolic pressure (RVSP), mean arterial pressure (MAP), and heart rate (HR) were measured under pentobarbital (60 mg/kg; i.p.) anesthesia and artificial respiration via a trachea cannula. The peak rate of right ventricular pressure (RV  *dP*/*dt*_max⁡_) and relaxation time constant (*τ*), as indices of myocardial contractility, were also calculated from RVSP. All parameters were continuously recorded using Powerlab system (AD Instruments, Australia).

### 2.4. Tissue Weights

Following the hemodynamic measurements, animals were sacrificed by an overdose of pentobarbital. The hearts and lungs were removed and weighed. Then, the tissues were cut into small pieces for drying at 55°C until a constant weight was reached. Wet-to-body weight ratios and wet-to-dry weight ratios of the tissues were calculated to assess the degree of the congestion [10]. The ratio of the wet weight of the RV to that of the LV + S  [RV/(LV + S)] was calculated as an indicator of RV hypertrophy.

## 3. Measurements of Blood Oxidants and Antioxidative Enzymes

The enzyme activities of CAT (Cayman, Chemical Company, MI), SOD, and GSH-Px (RANDOX Laboratories Ltd., UK) were determined by using spectrophotometric assay kits. Serum glutathione (GSH) and glutathione disulfide (GSSG) contents were measured using colorimetric enzymatic kits from Assay Designs/Stressgen Bioreagents. The MDA levels were measured spectrophotometrically based on the coupling of MDA with thiobarbituric acid [15].

### 3.1. Statistics

Data were presented as mean ± SE. Comparisons between groups were made with Student's paired *t*-test or one-way ANOVA as appropriate. If ANOVA analysis indicated significant differences, a Tukey's multiple-comparison posttest was performed to compare mean values between treatment groups and control. In addition to the ANOVA, a standard regression analysis for linear response in the dose groups was performed. A *P* value of <0.05 was considered statistically significant.

## 4. Results

### 4.1. Morphometric and Hemodynamic Evaluation

Table 1 shows a comparison of the morphometric and hemodynamic parameters of rats in the control group and the MCT groups. There were no significant differences in either LV + S or lung dry/wet ratio among groups. Compared to the control group, MCT administration caused a significant increase in lung wet and lung/body weight. The MCT-induced increase in lung/body weight was significantly attenuated by MSM in a dose-dependent manner (*P* < 0.01). The RV/LV + S ratio was calculated as an index of right ventricular hypertrophy. The value of RV/LV + S ratio was significantly higher in rats exposed to MCT, which was significantly reversed by MSM treatment (*r* ^2^ = 0.50; *P* < 0.01).

Levels of right ventricular systolic pressure (RSVP), peak rate of right ventricular pressure (RV  *dP*/*dt*_max⁡_), and relaxation time constant (*τ*) were significantly increased at week 4 of MCT treatment. All the hemodynamics parameters were significantly improved in all MSM-treated rats when compared with those in the saline-treated pulmonary hypertensive rats (Table 1).

### 4.2. Activities of Antioxidant Enzymes

Levels of CAT, SOD, and GSH-Px in the serum of hypertensive rats were significantly decreased at week 4 of MCT treatment (Table 2). The effects of MSM (0–400 mg/kg/day) given from the 10th day prior to MCT-induced hypertension on the activities of serum antioxidant enzymes are shown in Table 2. The activities of all CAT, SOD, and GSH-Px were significantly increased (*P* < 0.01) in MSM-treated rats. Both ANOVA and linear regression demonstrated a dose-proportional rise in activities of antioxidant enzymes in MCT-induced hypertensive rats that were treated with MSM.

### 4.3. Levels of GSH and GSSG

**Figure 1:**
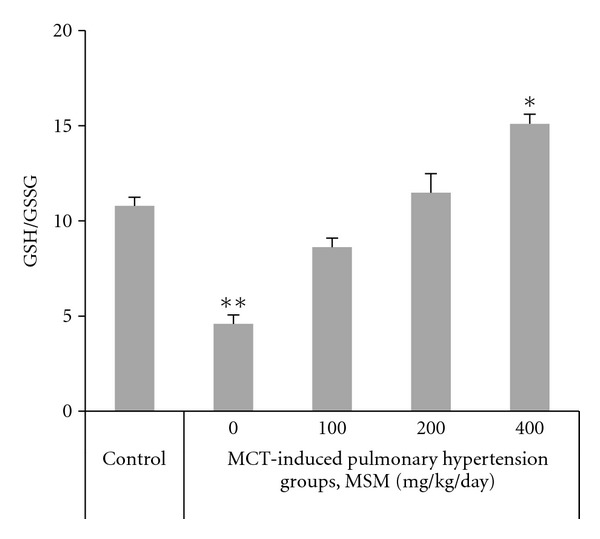
Effects of different doses (0–400 mg/kg/day) of methylsulfonylmethane (MSM) on the reduced to oxidized glutathione (GSH/GSSG) in rats with monocrotaline- (MCT-) induced pulmonary hypertension (regression coefficient *r* ^2^ = 0.84, *P* < 0.01). Data are means  ± SE, *n* = 6. Significant Tukey's post hoc differences compared with controls are indicated by asterisks (**P < 0.01 and *P < 0.05).

The effect of MSM treatment on the serum content of GSH and GSSG is shown in Table 3. MSM administration at 100 mg/kg/day significantly restored the levels of GSH and GSSG by +58% and −17%, respectively. The ratio of GSH/GSSG was calculated as an index of the redox state. The value of GSH/GSSG ratio was significantly lower in MCT-induced PAH (*P* = 0.002), but it was recovered to control levels after the treatment with 200 mg/kg/day MSM (Figure 1) and further enhanced by treatment with 400 mg/kg/day MSM (*r* ^2^ = 0.84; *P* < 0.01).

### 4.4. Level of Lipid Peroxidation

**Figure 2:**
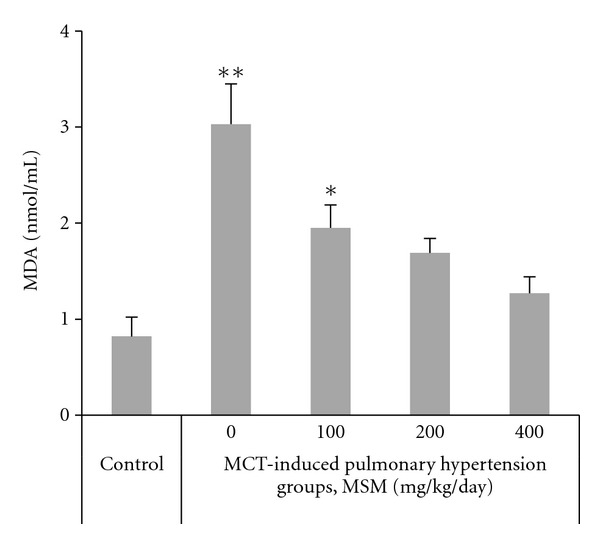
Effects of different doses (0–400 mg/kg/day) of methylsulfonylmethane (MSM) on lipid peroxidation expressed as the contents of malondialdehyde (MDA) in rats with monocrotaline- (MCT-) induced pulmonary hypertension (regression coefficient *r* ^2^ = 0.44, *P* < 0.01). Data are means  ± SE, *n* = 6. Significant Tukey's post hoc differences compared with controls are indicated by asterisks (**P < 0.01 and *P < 0.05).

The serum levels of MDA were assayed as index of lipid peroxidation. Levels of MDA in the serum of hypertensive rats were significantly increased versus control (Figure 2). In MCT-induced hypertensive rats, MSM treatment led to a significant decrease in serum MDA level. MSM caused a dose-dependent decrease in MDA, with a maximal suppression of 60% in response to 400 mg/kg/day MSM.

## 5. Discussion

In this study, significant changes in the hemodynamics, serum antioxidative enzymes, glutathione, and MDA were observed in MCT-induced pulmonary hypertensive rats, and these changes were modulated with MSM in the experimental treatments.

Our findings are in agreement with previous reports showing adverse effects on the hemodynamics and lower final body weight gain in rats treated with MCT [16]. In the animals that received MSM, the hemodynamics were maintained at control levels, indicating prevention from the effects of MCT.

Consistent with the current study, several studies have reported that cardiovascular system of MCT-induced pulmonary hypertensive rats showed inflammatory alterations similar to those observed in human PAH [17, 18]. The effects of PAH on myocardium include hypertrophy and interstitial inflammatory cell infiltration. The RV tissue in the rat model of PAH was generally characterized by oxidative stress generation and showed elevation in stress markers MDA and nitrotyrosine [18]. Oxidative events observed in patients with chronic heart failure included markedly increased serum levels of the MDA and decreased GSH-px [19].

Furthermore, the finding of increased RAAS and ET-1 in rats with MCT-induced pulmonary hypertension supports the presence of enhanced oxidative stress in the RV of PAH rats. The PAH-induced increase in RAAS and ET-1 was reverted by treatment of MSM, indicating an improvement in the oxidative status of the cells.

In this study, enzymatic activity assay showed reduced CAT, SOD, and GSH-Px in serum of PAH rats compared with control. Biphasic changes in antioxidant enzymes have been described in association with the time point of sampling during MCT-induced PAH. These changes include an increase in antioxidant enzymes during hypertrophy stage and a decrease during heart failure stage [20]. Overall concordance between changes in hemodynamics and codirectional changes in oxidative markers in the current study implicates a role of oxidative stress in the pathogenesis of PAH.

In this study, CAT and SOD activities were higher in 400 mg/kg/day MSM-treated pulmonary hypertensive rats than in either the normotensive controls or the MCT-induced pulmonary hypertensive rats. A similar observation was previously made by Jin et al. [21] who showed that CAT, SOD, and GSH-Px activities in the plasma increased obviously in the MCT-induced hypertensive rats with the use of a sulfur dioxide (SO2) donor. These results imply that the SO2 can, at least partly, increase the antioxidative capacity of rats. These results are consistent with previous reports showing antioxidative effects of MSM through inhibition of oxidant production [22].

Recent reports have implicated increased lipid peroxidation as a mediator in the pathogenesis and the development of PAH [23]. Accordingly, antilipid peroxidation treatment therapy has been effective in the treatment of pulmonary arterial pressure and pulmonary resistance in PAH [24]. The finding of decreased lipid peroxidation marker MDA in rats with MCT-induced pulmonary hypertension supports the presence of enhanced oxidative stress in the RV of PAH rats. The PAH-induced increase in MDA was reverted by treatment of MSM, indicating an improvement in the oxidative status of the cells. Kim et al. [22] have recently shown that MSM inhibits LPS-induced release of oxidative stress biomarkers such as nitric oxide and prostaglandin E2 in macrophages through downregulation of NF-*κ*B signaling. Moreover, MSM may act directly as free radical scavenger, which would further add to the efficiency of MSM as an antioxidant [7].

In pretreatment assessments, a decrease in circulating elevation of oxidative markers and an improved overall antioxidant potential with MSM treatment were evident. To the authors' knowledge, there are no specific data regarding the effect of MSM on oxidative parameters in pulmonary hypertensive rats. Although no mechanistic interpretation can be made at this point, the results obtained in the present study provide evidence for the first time that the MSM could limit oxidative response following pulmonary hypertension. Future studies regarding the effects of MSM treatment on antioxidative defense function in PAH are clearly warranted.

## 6. Conclusion

MSM could exert protective antioxidative effects through the induction of CAT, SOD, and GSH-px activities along with associated reducing agents, such as GSH. In addition, these results suggest that MCT-induced PAH could induce harmful effects on the RV function, probably due to a decrease in antioxidant enzyme activity and subsequent oxidative damage.

## Figures and Tables

**Table 1:** Morphometric parameters and hemodynamic data of control and monocrotaline-injected male rats.

	Control	MCT-induced pulmonary hypertension groups. MSM (mg/kg/day)					
		0	100	200	400	*r* ^2^	*P*
Morphometric parameters							
Body wt (g)	265 ± 8	204 ± 12*	230 ± 10	244 ± 21	262 ± 11	0.39	<0.01
Heart (g)	0.71 ± 0.13	0.81 ± 0.19	0.78 ± 0.18	0.75 ± 0.20	0.74 ± 0.17	0.09	0.21
Heart/body wt (mg/g)	2.67 ± 0.11	3.4 ± 0.14**	3.44 ± 0.27	3.23 ± 0.53	2.84 ± 0.054	0.36	<0.01
RV wt (g)	0.16 ± 0.01	0.31 ± 0.04**	0.25 ± 0.02	0.22 ± 0.03	0.18 ± 0.01	0.38	<0.01
RV/body wt (mg/g)	0.59 ± 0.04	1.54 ± 0.15**	1.09 ± 0.15*	0.92 ± 0.27	0.70 ± 0.38	0.46	<0.01
LV + S wt (g)	0.55 ± 0.02	0.50 ± 0.01	0.53 ± 0.02	0.53 ± 0.01	0.56 ± 0.02	0.23	0.04
LV + S/body wt (mg/mg)	2.08 ± 0.08	2.46 ± 0.08	2.33 ± 0.15	2.24 ± 0.26	2.15 ± 0.07	0.15	0.11
RV/LV + S	0.28 ± 0.02	0.62 ± 0.07**	0.47 ± 0.04*	0.42 ± 0.06	0.33 ± 0.03	0.50	<0.01
Lung wet (g)	1.11 ± 0.06	3.09 ± 0.14**	2.23 ± 0.36*	1.99 ± 0.26	1.28 ± 0.09	0.60	<0.01
Lung dry (g)	0.25 ± 0.01	0.52 ± 0.02**	0.46 ± 0.06*	0.41 ± 0.06	0.27 ± 0.02	0.50	<0.01
Lung dry/wet	0.22 ± 0.03	0.17 ± 0.02**	0.20 ± 0.09	0.21 ± 0.03	0.21 ± 0.03	0.36	0.01
Lung wet/body wt (mg/g)	4.19 ± 0.18	15.17 ± 1.15**	9.35 ± 2.06*	8.17 ± 2.38	4.83 ± 0.20	0.60	<0.01
Hemodynamic data							
HR (beats/min)	397 ± 31	274 ± 19*	312 ± 16	326 ± 24	406 ± 31	0.47	<0.01
MAP (mmHg)	83.28 ± 7.42	54.07 ± 7.13**	53.100 ± 2.76**	58.68 ± 3.149*	77.83 ± 1.31	0.62	<0.01
RVSP (mmHg)	22.56 ± 1.65	35.87 ± 1.76**	29.21 ± 2.79	27.65 ± 2.60	22.62 ± 2.11	0.53	<0.01
RV *dP*/*dt*_max⁡_ (mmHg/s)	1958 ± 40	2376 ± 116*	2196 ± 25	2130 ± 50	2027 ± 39	0.39	<0.01
*τ* (ms)	14.43 ± 0.92	22.62 ± 0.83**	19.05 ± 1.43*	17.44 ± 0.91	15.65 ± 0.97	0.53	<0.01

Data are mean ± SE. Significant Tukey's post hoc differences compared with controls are indicated by asterisks (**P < 0.01 and *P < 0.05) and *r* ^2^ is the regression coefficient of the dose-response effect. MCT: monocrotaline; MSM: methylsulfonylmethane; RV: right ventricle; LV: left ventricle; S: septum; wt: weight; HR: heart rate; MAP: mean arterial pressure; RVSP: right ventricular systolic pressure; RV *dP*/*dt*_max⁡_: peak rate of right ventricular pressure; *τ*: relaxation time constant.

**Table 2:** Effects of different doses of methylsulfonylmethane (MSM) on the activities of antioxidant enzymes in rats with monocrotaline-(MCT-) induced pulmonary hypertension.

	Control	MCT-induced pulmonary hypertension groups, MSM (mg/kg/day)					
		0	100	200	400	*r* ^2^	*P*
CAT (U/mL)	6.10 ± 0.83	2.02 ± 0.33*	4.16 ± 0.57	5.02 ± 0.75	7.98 ± 1.68	0.48	<0.001
SOD (U/mL)	203.33 ± 3.80	168.33 ± 6.67**	206.67 ± 5.11	210.00 ± 7.30	220.83 ± 3.96	0.50	<0.001
GSH-px (U/mL)	10.01 ± 0.12	7.05 ± 0.62**	8.14 ± 1.09	9.80 ± 0.25	10.20 ± 0.20	0.40	0.001

Significant Tukey's post hoc differences compared with controls are indicated by asterisks (**P < 0.01 and *P < 0.05) and *r* ^2^ is the regression coefficient of the dose-response effect. MCT: monocrotaline; MSM: methylsulfonylmethane; SOD: superoxide dismutase; GSH-Px: glutathione peroxidase; CAT: catalase; GSH: reduced glutathione; GSSG: oxidized glutathione; MDA: malondialdehyde.

**Table 3:** Effects of different doses of methylsulfonylmethane (MSM) on the levels of reduced glutathione (GSH) and disulfide-oxidized glutathione (GSSG) and MDA in rats with monocrotaline- (MCT-) induced pulmonary hypertension.

	Control	MCT-induced pulmonary hypertension groups, MSM (mg/kg/day)					
		0	100	200	400	*r* ^2^	*P*
GSH (nmol/mL)	543.54 ± 16.22	347.33 ± 41.54*	551.65 ± 93.28	695.95 ± 106.36	774.62 ± 45.62	0.41	<0.01
GSSG (nmol/mL)	50.71 ± 2.17	76.19 ± 6.51*	62.98 ± 9.36	59.05 ± 4.80	51.31 ± 2.41	0.26	0.01

Significant Tukey's post hoc differences compared with controls are indicated by asterisks (**P < 0.01 and *P < 0.05) and *r* ^2^ is the regression coefficient of the dose-response effect. MCT: monocrotaline; MSM: methylsulfonylmethane; SOD: superoxide dismutase; GSH-Px: glutathione peroxidase; CAT: catalase; GSH: reduced glutathione; GSSG: oxidized glutathione; MDA: malondialdehyde.
